# Bioactivity of microbial biofilms in extreme environments

**DOI:** 10.3389/fmicb.2025.1602583

**Published:** 2025-09-05

**Authors:** Shriya P. Bhat, David J. Roach

**Affiliations:** ^1^Department of Molecular and Cellular Biology, Harvard University, Cambridge, MA, United States; ^2^Broad Institute, Cambridge, MA, United States; ^3^Division of Infectious Diseases, Brigham and Women’s Hospital, Boston, MA, United States

**Keywords:** biofilms, bioactivity, extreme environments, EPS, extremophile

## Abstract

Biofilms, which are highly structured microbial communities encased in a self-produced matrix, are frequently employed by many bacteria and archaea with significant implications for their survival in extreme environments. These environments, characterized by extreme temperatures, pH, salinity, and variable nutrient availability, can pose challenges that biofilms help organisms overcome through unique adaptations. This review explores the bioactivity of biofilms in extreme environments, highlighting biofilms’ ability to produce novel biomolecules and other biofunctions with potential applications in medicine and biotechnology. Key adaptations such as extracellular polymeric substances, cooperative and competitive interactions, and specialized nutrient acquisition strategies are examined for their roles in biofilm resilience and bioactivity. The potential of these biofilms to contribute to the development of novel therapeutics, antimicrobial agents, antioxidants, and anticancer compounds is discussed, underscoring their significance in advancing medical and biotechnological applications. Through an in-depth analysis of current knowledge, this review highlights the bioactive capacities of extremophilic biofilms and their promising applications for human benefit.

## 1 Introduction

**Figure 1:**
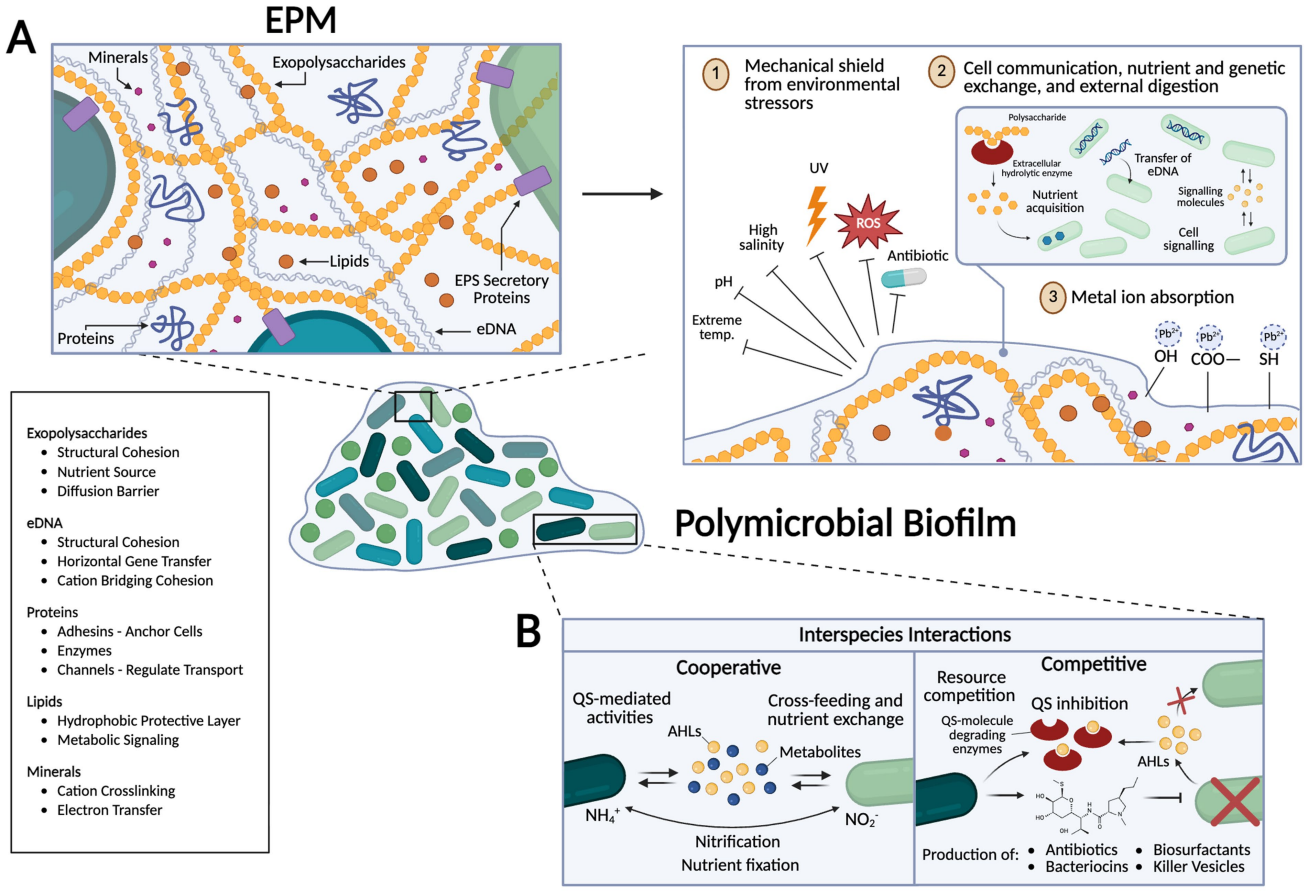
Schematic representation of biofilm features and their role in microorganisms’ ability to withstand extreme environments. **(A)** The Extracellular Polymeric Matrix (EPM) consists of polysaccharides, lipids, proteins, minerals, and extracellular DNA (eDNA). The EPS matrix provides structural support and acts as a mechanical barrier to environmental stressors such as extreme pH, salinity, radiation, reactive oxygen species (ROS). Within this matrix, EPS facilitates cell-to-cell communication, nutrient exchange, and genetic transfer of eDNA and plasmids (horizontal gene transfer) by increasing effective concentration. Extracellular hydrolytic enzymes break down complex molecules for nutrient acquisition, a particularly useful adaptation in nutrient-deficient environments. Functional groups within the EPS, including hydroxyl, carboxyl, and sulfhydryl groups, can bind and sequester metal ions, potentially aiding in detoxification or mineral acquisition. **(B)** Polymicrobial biofilms exhibit both cooperative and competitive interspecies interactions. Cooperative interactions include Quorum Sensing (QS)-mediated activities, cross-feeding (where different genotypes or species exchange different metabolites), and nutrient fixation processes (e.g., nitrification) where produced metabolites are directly exchanged between species to minimize loss and increase effective substrate use. Competitive interactions include resource competition and production of antibiotics, bacteriocins, biosurfactants, and killer vesicles to outcompete other microorganisms. QS inhibition can also disrupt communication in competing species. Created in https://BioRender.com.

It is estimated that up to 80% of bacterial and archaeal cells exist in the form of biofilm (Bar-On and Milo, 2019; Flemming and Wuertz, 2019). Microbial biofilms, which are communities of microorganisms adhering to living or inert surfaces, are encased in a self-produced matrix of extracellular polymeric substances (EPS), including proteins, polysaccharides, lipids, nucleic acids, and extracellular DNA (eDNA) (Flemming and Wuertz, 2019) (Figure 1). These communities are highly structured, with distinct microenvironments and adaptations that support survival in nearly every environmental condition on Earth (Wong and O’Toole, 2011; Vestby et al., 2020).

Of growing interest in evolutionary biology and biomedicine is the existence of microbial life in extremophilic conditions—environments generally considered uninhabitable for most biological life due to extreme temperature, pH, salinity, nutrient scarcity, and toxic waste (Parrilli et al., 2022; Yin et al., 2019). The cyclical development of biofilm, characterized by repeated cycles of attachment, growth, maturation, dispersal, and reattachment, enables their adaptation and persistence in such conditions, producing a physically distinct habitat that offers protection from extreme environmental factors (Parrilli et al., 2022; Baker-Austin et al., 2010).

Due to the versatility and persistence of biofilm communities in extreme environments, there is a growing interest in their activity—the ability of organisms to produce novel biomolecules or generate other useful biofunctions. If better understood, certain adaptations—most notably the synthesis of unique antimicrobial and antioxidant metabolites—could contribute to developing novel therapeutics to combat disease (Knight et al., 2003). In this narrative review, we explore the current literature of the bioactivity of biofilm communities in extreme environments, summarizing their key adaptations contributing to their survival and their potential applications in medicine, biotechnology, and the environment. We organize the review by major bioactivity classes (antimicrobials, antioxidants, cryoprotectants, anticancer agents, and bioremediation compounds). Finally, a discussion synthesizes the current state of the field, knowledge gaps, and future directions for harnessing extremophilic biofilms in biotechnology.

## 2 Biofilm adaptations in extreme environments

Biofilms have been discovered in nearly all environments on Earth, including deep-sea hydrothermal vents, geothermal hot springs, hypersaline inland seas, and frozen Antarctic glaciers (Edwards et al., 2012; Karley et al., 2019; Smith et al., 2016). Some of the earliest evidence of their existence include fossils from a 3.2-billion-year-old deep-sea volcanic deposit in the Pilbara region of Australia and similar-aged hydrothermal sediments in the Barberton greenstone belt of South Africa (Smith et al., 2016). This suggests that biofilm formation may be a protective feature of early prokaryotes, shielding microorganisms from conditions otherwise intolerant to most biological life—and that the genetic and molecular mechanisms required for biofilm formation were already established at this early stage in evolution (Yin et al., 2019; Westall et al., 2001; Rasmussen, 2000). An overview of the key structural and functional adaptations that enable biofilm survival is presented in Figure 1.

### 2.1 Extracellular polymeric matrix

Perhaps the most ubiquitous and useful adaptation of biofilm is their extracellular polymeric matrix (EPM), a network of extracellular macromolecules produced by bacteria, which shields them from their outside environment (Costa et al., 2018; Flemming et al., 2007; Pan et al., 2016). Along with providing structural support to maintain biofilm integrity, EPM broadly facilitates cell-to-cell signaling, providing an environment for exchange of quorum sensing molecules such as acyl-homoserine lactones (AHLs) in *Pseudomonas aeruginosa*, which coordinate collective virulence and biofilm formation (Flemming et al., 2007). EPM also provides detoxification and protection against diverse stressors, such as extreme temperature, salinity, pH, and low nutrient availability (D’Urzo et al., 2014; Mahto et al., 2022; Blanco et al., 2019). For example, inositol and 3-O-methylglucose sugars commonly found in the EPM are critical in mitigating oxidative stress and heavy-metal toxicity, whereas high eDNA and sugar content in cold environments function as cryoprotectants (Blanco et al., 2019).

Extremophilic biofilms, and bacteria isolated from biofilms in extreme conditions, have uniquely adapted to such conditions through the production of specialized extracellular polymeric substance (EPS) compositions—including uronic acid-rich EPS with metal-chelating properties, sulfated EPS with antioxidant and cryoprotective activities, and glycine-rich polysaccharides that prevent ice crystal formation—that not only enhance survival but also contribute to their remarkable bioactivity (Banerjee et al., 2021; Nagar et al., 2021; Sarkar et al., 2024). In thermophilic environments, extremophilic biofilms produce thermostable EPSs that maintain structural cohesion and mediate ion exchange under high temperature and acidity (Sarkar et al., 2024; Zhang et al., 2019). *Acidianus* sp. DSM 29099, an obligate thermoacidophile, forms biofilms on pyrite at 70 °C and pH ~ 2. Its EPS contains mannose, glucose, fucose, and uronic acids, which likely facilitate adhesion to mineral surfaces and metal ion sequestration—critical for survival and bioleaching activity in acidic geothermal niches (Zhang et al., 2019). In cold environments, EPS composition shifts to confer cryoprotective functions, the major component being exopolysaccharides with a glass transition temperature (T_g_)—the temperature at which the structure transitions from a “rigid” to a “flexible” state—of −20 °C, which lies at least several tens of degrees lower than the average exopolysaccharide T_g_ in non-extremophilic contexts (Nagar et al., 2021; Inomata et al., 2025; Freitas et al., 2009). Sea-ice-associated Antarctic bacteria secrete polysaccharides to form viscous biofilms, preventing ice crystal formation and cellular desiccation. EPS produced by *Pseudoalteromonas* sp. from Antarctic Sea ice forms a protective matrix around cells and significantly enhances survival under freezing conditions (Nagar et al., 2021). Similarly, sulfated and uronic acid-rich EPS from Antarctic cyanobacteria act as natural antifreeze agents, which can trap nutrients and offer UV protection (often pigmented) (Nagar et al., 2021). While specific compositions vary, common Antarctic EPS types include alginate polysaccharides derived from glucose and mannose and xanthan gum analogs rich in glucuronic acid and sulfate groups (Nagar et al., 2021; Casillo et al., 2017). Notably, these cold-adapted compositions provide functionality beyond cryoprotection due to their polyanionic nature, exhibiting radical scavenging capabilities comparable to ascorbic acid, as found by a α-mannan exopolysaccharide from Arctic *Sphingobacterium* (Chatterjee et al., 2018). Furthermore, *Pseudoalteromonas* sp. MER144, isolated from Antarctic seawater, produces a high-molecular-weight (~250 kDa) EPS composed primarily of glucose, mannose, galactosamine, and uronic acids, with notable levels of uronic acids (14%), proteins (12%), and sulfates (3.1%) (Caruso et al., 2017). Alongside conferring cryoprotection, improving cell viability by up to 50% after repeated freeze–thaw cycle, the EPS demonstrates strong cadmium chelation (up to 48% removal in 60 min) and enhanced production under mercury and cadmium stress (Caruso et al., 2017). Similarly, *Marinobacter* sp. W1-16 produces a sulfate- and uronic-acid-rich EPS that demonstrates emulsifying and metal-binding activity (Caruso et al., 2019).

In acidic, metal-laden systems, chemolithoautotrophs such as *Acidithiobacillus ferrooxidans* form EPS-enriched biofilms on sulfide minerals that anchor cells in place while regenerating Fe^3+^ oxidants (Moncayo et al., 2022). This process is already taken advantage of in industrial biooxidation and bioleaching, which depend on acidophilic biofilms to break down sulfide minerals and regenerate oxidizing agents to extract metals from low-grade or refractory ores (Marques, 2018). Indeed, the EPS of acidophilic biofilms are typically rich in uronic acids and proteins, which aid in mineral attachment and act as local buffers against low pH (Moncayo et al., 2022). Moncayo et al. found that under high Fe^3+^ (18 g L^−1^) concentrations and low galactose (0.15%), EPS synthesis of *A. ferrooxidans* was upregulated, improving biofilm attachment to refractory polymetallic sulfide ore from 71 to 94% compared to no galactose treatment (2022). This suggests that *A. ferrooxidans* EPS could act as a tunable target that can be further optimized to be produced beyond its native levels for industrial bioleaching, with the potential to shorten leach cycles, increase metal recovery, and reduce reliance on costly chemical oxidants.

Finally, halophilic archaea in hypersaline environments, such as *Haloarcula hispanica*, produce large, acidic EPS composed predominantly of mannose and galactose (Lü et al., 2017). These polymers are essential for osmotic balance, biofilm formation, and protection against desiccation. EPS-deficient mutants of *H. hispanica* exhibit impaired growth under salt stress and altered cell surface properties, underscoring the role of EPS as a hydrated barrier in extreme salinity (Lü et al., 2017).

In addition to these protective and structural functions, the EPM plays a key role in promoting horizontal gene transfer (HGT) (Flemming and Wuertz, 2019). The close spatial arrangement of cells within the matrix, along with the abundance of eDNA, creates a microenvironment conducive to transformation, conjugation, and transduction. This promotes rapid acquisition of adaptive traits, including stress resistance and metabolic versatility—critical for survival under extreme conditions (Flemming et al., 2007; Flemming and Wuertz, 2019; Kobras and Falush, 2019). Overall, extremophilic biofilm matrices are rich in EPS (sugars, proteins, eDNA) that serve as a physical shield and biochemical buffer, enabling life in extremes (Méndez-García et al., 2015; Nagar et al., 2021). The matrix can scavenge reactive oxygen species generated by thermal or UV stress, bind or precipitate toxic metals in acid mine drainage, and retain water in deserts or salt flats, illustrating its central role in mediating microbial survival and ecological function under extreme environmental pressures (Adessi et al., 2018; Sarkar et al., 2024; Chatterjee et al., 2018).

#### 2.1.1 Extraction methods for extracellular polymeric substances

**Figure 2:**
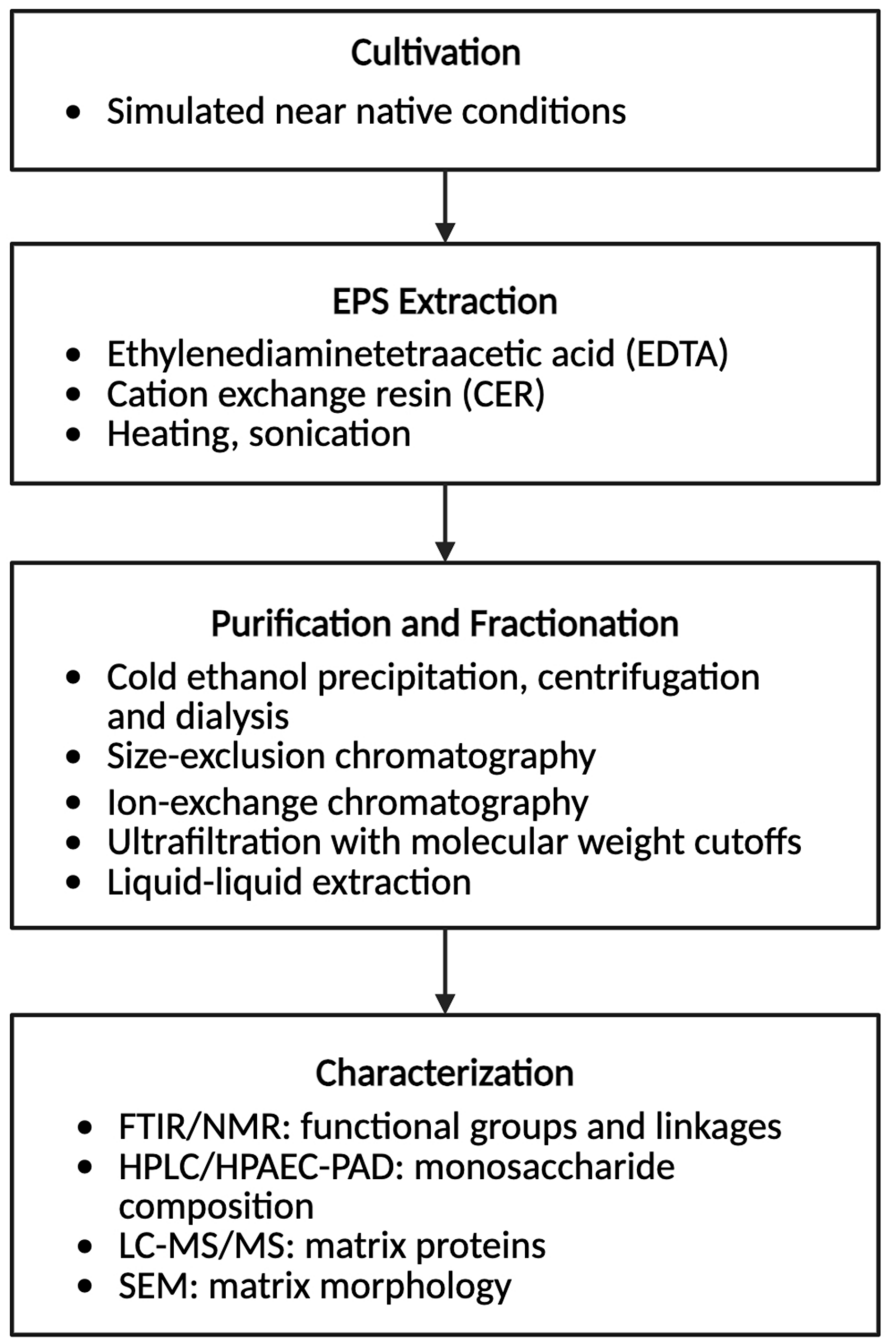
Workflow for extraction and characterization of extracellular polymeric substances (EPS) from extremophilic biofilms. Created in https://BioRender.com.

Given the central role of EPS in shaping the structure, resilience, and bioactivity of extremophilic biofilms, methods for their extraction and characterization are critical to advancing both fundamental understanding and translational applications. The isolation and characterization of EPS in laboratory settings typically begins by cultivating the organism under near native simulated conditions (Figure 2). Common extraction methods for EPS involve high-salt buffers to maintain solubility and disrupt electrostatic interactions, ethylenediaminetetraacetic acid (EDTA) or cation-exchange resins to sequester divalent cations, or mild heating in thermophiles to reduce viscosity and facilitate polymer release (Casillo et al., 2018; Caruso et al., 2017; Kang et al., 2024). The EPS is then recovered from the cell-free supernatant by cold-ethanol precipitation followed by centrifugation and dialysis or tangential flow filtration to remove salts, solvents, and low-molecular-weight impurities (Casillo et al., 2018). The crude EPS can be further fractionated using size-exclusion chromatography, ion-exchange chromatography, ultrafiltration with defined molecular weight cutoffs, or three-phase partitioning to help resolve individual biopolymer classes (Jachlewski et al., 2015; Antunes et al., 2024). For lipid-rich matrices, solvent-based extraction (e.g., modified Bligh and Dyer or Folch methods) is employed, sometimes with sonication or heating to enhance recovery of long-chain or ether-linked lipids (Krivoruchko et al., 2025; Li et al., 2025). EPS characterization can involve Fourier-transform infrared (FTIR) spectroscopy and nuclear magnetic resonance (NMR) spectroscopy to identify functional groups and linkages; High-performance liquid chromatography (HPLC) or High-performance anion-exchange chromatography with pulsed amperometric detection (HPAEC-PAD) to determine monosaccharide composition; SDS-PAGE, two-dimensional gel electrophoresis; LC–MS/MS proteomics to identify matrix-associated proteins; and scanning electron microscopy (SEM) to visualize matrix morphology (Caruso et al., 2017; Gieroba et al., 2020; Pornsunthorntawee et al., 2008) (Figure 2).

### 2.2 Cooperative interactions

Extremophiles often live in multispecies biofilms where metabolic cooperation is crucial for survival, including exchanging nutrients and metabolic byproducts (Madsen et al., 2016). On desert rocks, multispecies biofilms exhibit synergistic interactions between cyanobacteria and fungi. Cyanobacteria supply essential nutrients to fungi, while the fungi release vital metals from the rock that benefit the cyanobacteria (Gorbushina and Broughton, 2009).

In acid mine drainage (AMD) biofilms (pH < 2), microbial communities exhibit niche partitioning and resource sharing that support survival in highly acidic, nutrient-poor conditions (Méndez-García et al., 2015). *Ferrovum myxofaciens*, an iron-oxidizing acidophile, frequently dominates AMD streamer biofilms, producing EPS and fixing atmospheric nitrogen to support surrounding community members (Méndez-García et al., 2015). Genomic analyses have revealed a complete *nif* gene cluster for nitrogen fixation, as well as expression of carbon fixation pathways, suggesting that *Ferrovum* serves as a primary producer supplying organic carbon and nitrogen to heterotrophic partners (Méndez-García et al., 2015). These cooperative interactions establish a stable consortium in which chemolithotrophs generate biomass that sustains other extremophiles within the biofilm. Similar trophic cooperation has been observed in deep-sea hydrothermal vent biofilms, where diverse bacteria and archaea co-exist across steep chemical gradients (Ladd et al., 2024). Sulfur-oxidizing bacteria residing on vent chimneys fix carbon dioxide and secrete organic compounds that are subsequently utilized by hydrogen-oxidizing and heterotrophic archaea, forming tightly interdependent metabolic networks. These mutualistic exchanges—including nutrient sharing, communal EPS production, and physiological co-adaptation—enhance biofilm resilience in extreme environments.

Furthermore, recent metagenomic studies of extreme microbiomes suggest extensive metabolic crosstalk and co-regulation within biofilms (Peng et al., 2024; Galvez et al., 2022; Van et al., 2017). In copper mining tailings, which are characterized by low pH and high concentrations of heavy metals, *Flavobacteria*, *Pseudomonas*, and *Erwinia* exist in highly coordinated biofilms, with *Flavobacteria* producing secondary metabolites that can mitigate oxidative stress, *Erwinia* contributing to metal bioleaching and reduction, and *Pseudomonas* enhancing resistance by modulating local metal concentrations (Galvez et al., 2022). Notably, *Flavobacteria* and *Erwinia* represented only a small fraction (1–8%) of the observed abundance, highlighting the importance of rare taxa within extremotolerant biofilms. Similarly, 16S rRNA-based co-occurrence network analysis of hypolithic biofilms in the hyperarid Namib Desert revealed that Cyanobacteria (e.g., *Pseudanabaenales*, *Oscillatoriales*) and Alphaproteobacteria (e.g., *Rhodobiaceae*, *Beijerinckiaceae*) form the backbone of active microbial networks, species that are known sources of phototrophy, nitrogen fixation, and metabolite exchange, suggesting these functions underpin microbial survival in nutrient-poor desert niches (Van et al., 2017). Once many of the network’s central nodes were low abundance taxa yet exhibited strong positive interdependencies with both heterotrophs and phototrophs, suggesting they play disproportionately large functional roles in nutrient cycling and energy transfer within the hypolithic niche (Van et al., 2017). Together, these findings highlight that metabolic cooperation within communities, often driven by rare but functionally pivotal taxa, is a fundamental adaptation for extremophiles to thrive as a community where no single species could survive alone.

Recognizing the robustness and efficiency of such interactions has prompted growing interest in leveraging extremophilic consortia for applied purposes, including biomining. Acidophilic bacteria and archaea—primarily *Acidithiobacillus*, *Leptospirillum*, *Sulfobacillus*, and *Ferroplasma* spp.—form biofilms on mineral sulfides and coordinate iron and sulfur oxidation through quorum sensing and metabolic exchange (Orell et al., 2010). Within these consortia, iron-oxidizers regenerate ferric iron while sulfur-oxidizers produce sulfuric acid, collectively enhancing metal solubilization and facilitating the recovery of copper, gold, nickel, and zinc (Marques, 2018; Orell et al., 2010). Industrial-scale heap leaching and stirred-tank biomining reactors exploit these interactions, operating at low pH (<2) and elevated temperatures (up to 80 °C) to prevent contamination and maximize efficiency. Up to 20% of global copper production now relies on such systems (Orell et al., 2010). Beyond mining, cooperative extremophilic biofilms have been applied to the remediation of metal-contaminated environments (N̆ancucheo and Johnson, 2011). Inoculated consortia consisting of iron-reducing bacteria (*Acidiphilium*, *Acidocella*), sulfate-reducing bacteria (*Desulfosporosinus*, *Desulfitobacterium*), and acidophilic algae (*Euglena*, *Chlorella*) facilitated pH buffering and metal immobilization through coupled redox processes. Algal-derived organic carbon sustained heterotrophic metabolisms, enabling dissimilatory iron and sulfate reduction that precipitated metals such as copper and zinc as sulfides (N̆ancucheo and Johnson, 2011). Other groups have developed communities to remediate petroleum hydrocarbons, engineering a halo thermo alkaliphilic consortia of *Marinobacter*, *Ochrobactrum*, *Pseudomonas*, and *Bacillus* spp., which achieved >90% degradation of polycyclic aromatic hydrocarbons in refinery wastewater at 60 °C, 8% salinity, and pH 10 (Al-Mur et al., 2021). Cooperative interactions including surfactant-mediated emulsification, syntrophic hydrocarbon metabolism, and mutual detoxification of reactive intermediates enabled substrate partitioning across strains and accelerated breakdown of both low- and high-molecular-weight hydrocarbons (Al-Mur et al., 2021). These studies underscore how cooperative interactions enable extremophilic consortia not only to thrive in inhospitable environments but also to be harnessed for real-world applications in resource recovery and environmental remediation.

### 2.3 Competitive interactions

In contrast with environments where cooperative interactions may be common, extreme environments—which may be characterized with steep physicochemical gradients and limited gas exchange—could favor competitive behaviors that regulate interspecies interactions due to spatial constraints, fluctuating or extreme chemical conditions, or highly specialized niches (Stewart, 2003; Penesyan et al., 2021; Rendueles and Ghigo, 2012). One mechanism of interest is the production of antimicrobial compounds by one species against another to protect microbial communities against unwanted invaders, an adaptation that might be particularly useful in oligotrophic conditions (Penesyan et al., 2021; Armbruster et al., 2016; Yin et al., 2019).

In hypersaline lakes and salterns, halophilic archaea secrete halocins—protein antibiotics effective against other haloarchaea (Martínez et al., 2022). For instance, *Haloferax mediterranei* biofilms secrete Halocin H1, a 31 kDa protein that disrupts the Na^+^/H^+^ balance in target cells, leading to cell lysis (Platas et al., 2002). Similarly, thermoacidophilic archaea of the genus *Sulfolobus* produce sulfolobicins, antimicrobial proteins that inhibit closely related strains lacking specific immunity (Gristwood et al., 2012). *Sulfolobus acidocaldarius*, for example, encodes two small sulfolobicin peptides, *SulA* and *SulB*, which are secreted and associated with membrane vesicles (Gristwood et al., 2012). These peptides specifically target and inhibit other *Sulfolobus* strains, thereby enabling the producer to monopolize surface attachment sites on mineral sulfur substrates (Gristwood et al., 2012; Ellen et al., 2011). Deletion of the sulfolobicin genes eliminates this inhibitory capacity, confirming their role in contact-dependent antagonism (Gristwood et al., 2012).

Competitive biochemical interactions are prevalent in extreme environments: biofilm dwellers utilize bacteriocins, archaeocins, and secondary metabolites to outcompete neighbors, ensuring access to nutrients and space. This microbial competition drives the evolution of potent bioactive compounds, including novel antibiotics, antifungals, and anticancer agents that could have therapeutic potential in medicine. The specific bioactive compounds produced through these interactions are examined in greater detail in the following sections.

## 3 Bioactive compounds of biofilms in extreme environments

Due to their unique adaptations, biofilm communities in extreme environments represent a promising source of novel biomolecules with unique functional activities (Chen et al., 2019; Li et al., 2017; Su et al., 2016) (Table 1). Due to their nutrient sequestering activities, biofilms have also been extensively studied for their remediation of contaminated soils, wastewater treatment systems, and deep-sea hydrothermal vents as well as production of novel antimicrobial compounds, enzymes, and secondary metabolites (Limoli et al., 2015; Biswal and Malik, 2022; Saini et al., 2023; de Lurdes and Enes Dapkevicius, 2012). Biofilm reactors have been effectively utilized to synthesize various high-value products, including antibiotics, bacteriocins, and other important enzymes and organic acids (Ercan et al., 2015). While not all source organisms described below can be strictly described as extremophilic, many are derived from environments with intense selective pressures that drive extremotolerant physiologies and unique biosynthetic pathways.

**Table 1:** Summary of bioactive compounds produced from biofilm lifestyles in extreme environments.

Activity	Compound	Microbial host	Extremal parameter	Key findings	Environment source	Reference
Antimicrobial	Halocin H1	*Haloferax mediterranei*	High salinity	Disrupts Na^+^/H^+^ balance, lyses competitor haloarchaea	Hypersaline lake/saltern	Platas et al. (2002)
	Sulfolobicins (*SulA*, *SulB*)	*Sulfolobus acidocaldarius*	High temperature (70 °C), low pH (~2)	Contact-dependent inhibition of related strains	Acidic hot spring	Gristwood et al. (2012)
	Huascopeptin-1 (lasso peptide)	*Streptomyces huasconensis*	High UV, aridity, salinity	Potent activity vs. *Bacillus subtilis*; thermostable	Atacama salt-flat spring	Pardo-Esté et al. (2024)
	Kribbellichelins A & B	*Kribbella* sp.	High salinity	Broad activity against human pathogens	Spanish saline wetland	Virués-Segovia et al. (2022)
	Cold-Azurin	Antarctic *Pseudomonas* sp.	Low temperature	Inhibits *S. epidermidis* biofilm on surfaces	Antarctic glacier	D’Angelo et al. (2024)
	EPS-coated AgNPs	*Pseudomonas* sp. PFAB4	Moderate thermophily	Antimicrobial vs. Gram-negatives & fungi	Thermal hot spring	Banerjee et al. (2019)
Antioxidant	Bacterioruberin	*Haloferax* spp.	High salinity & UV	~10 × vitamin E ROS scavenging	Solar saltern	Giani et al. (2023) and Hwang et al. (2024)
	DeinoPol (EPS)	*Deinococcus radiodurans*	High radiation	Reduces UV-B-induced ROS in keratinocytes	Nuclear lab waste	Lin et al. (2020)
	α-mannan EPS	*Sphingobacterium* sp. IITKGP-BTPF3	Low temperature	Superior superoxide scavenging	Arctic soil	Chatterjee et al. (2018)
	Sulfated EPS	Haloarchaeal strain	High salinity	Hydroxyl/superoxide radical scavenger	Saline brine	Chouchane et al. (2020)
Anticancer	Bacterioruberin extract	*Haloferax mediterranei*	High salinity & UV	Selective cytotoxicity to TNBC cells	Solar saltern	Giani et al. (2023)
	Cold-adapted azurin peptide	Antarctic *Pseudomonas* sp.	Low temperature	p53 stabilization, antitumor activity	Antarctic marine	D’Angelo et al. (2024) and Hu et al. (2023)
	Metabolite suite	*Nonomuraea* sp. PT708	Cave oligotrophy	Selective cytotoxicity toward lung/oral cancer lines	Thai cave soil	Nakaew et al. (2009)
	EPS fraction	*Streptomyces* sp. A5	Heavy-metal stress	Cytotoxic vs. breast & colon cancer cells	Contaminated soil	Homero et al. (2021)
Cryoprotectant	EPS (RosPo-2)	*Pseudoalteromonas* sp. RosPo-2	Low temperature	Doubles keratinocyte viability post-LN₂ freeze	Antarctic marine	Kang et al. (2024)
	EPS	*Pseudomonas* sp. BGI-2	Low temperature	Reduces RBC lysis, enhances bacterial freeze survival	Batura Glacier	Ali et al. (2020)
	Ectoine/Hydroxyectoine	*Halomonas elongata*	High salinity	Stabilizes proteins/membranes; medical osmo-protectant	Salt marsh	Sun et al. (2012) and Chen et al. (2023)
Bioremediation	EPS-mediated Cr(VI) reduction	*Lysinibacillus mangiferihumi*	High alkalinity & chromate	Reduces Cr(VI) → Cr(III), binds metal	Alkaline chromate soil	Tambekar et al. (2015)
	Sulfide mineral precipitation	Sulfate-reducing bacteria consortium	High pH (~11)	Immobilizes 90Sr & 137Cs as sulfides	Nuclear waste leachate	Ruiz-Fresneda et al. (2023)
	EPS emulsifier	*Pseudomonas furukawaii*	High salinity	Degrades 89.5% crude oil in 5 days	Marine spill simulation	Vandana and Das (2021)
	Uranium biosorption (EPS)	*Deinococcus radiodurans*	High radiation	Sequesters 90% U(VI) as U(IV)	Radioactive wastewater	Manobala et al. (2021)
	EPS-enhanced bioleaching	*Acidithiobacillus ferrooxidans*	Low pH, high metals	Improves metal chelation & sulfide ore oxidation	Acid-mine drainage	Moncayo et al. (2022)
	Sulfate- and uronic-acid-rich EPS	*Marinobacter* sp. *W1-16*	Low temperature	Emulsifying and metal-binding activity	Antarctic seawater	Caruso et al. (2019)

### 3.1 Antimicrobials

Bacteria have spent billions of years evolving chemical defenses to compete with neighboring microbes, and modern medicine has capitalized on this natural arms race to develop a wide array of antimicrobial agents. Actinobacteria and bacteria of the genus *Streptomyces* are responsible for producing approximately 45–80% of all natural bioactive compounds with pharmacological potential, while social microbes account for two-thirds of all commercially available antibiotics (Banerjee and Joshi, 2013; Barka et al., 2015; Rangseekaew and Pathom-aree, 2019; Quinn and Dyson, 2024).

Despite this rich history of drug discovery, the rate of new antimicrobial discovery has slowed, and novel bioactive compounds are increasingly presumed to arise from previously untapped ecosystems (Cardona et al., 2025). Biofilm lifestyles, particularly in extreme environments, appear to promote antimicrobial production through mechanisms such as interspecies competition and quorum-sensing-dependent regulation of secondary metabolism, which ensure metabolic efficiency only at high cell densities (Ghosh et al., 2017; Baranova et al., 2020). In hypersaline environments, biofilm-forming haloarchaea produce proteinaceous antimicrobials with remarkable stability (Dutta and Bandopadhyay, 2022; Chen et al., 2019). Halocins, secreted by *Haloferax* and *Halobacterium* species, retain activity at saturating salt concentrations that typically inactivate conventional antibiotics (Chen et al., 2019). It is long known that Halocin H6 from *Hfx. gibbonsii* inhibits *Halobacterium* by targeting its Na^+^/H^+^ antiporter, a mechanism of action specific to high-salt conditions (Torreblanca et al., 1990; Meseguer et al., 1995). Smaller “microhalocins” (<10 kDa), such as HalS8—a hydrophobic peptide—are also noteworthy for their resistance to boiling, proteolysis, and pH extremes (Price and Shand, 2000). Though primarily active against other archaea, these extremophile-derived antimicrobials expand the known repertoire of heat- and salt-stable antibiotics and may prove valuable for controlling biofilms in industrial or food-processing environments. Acidophilic extremophiles have also yielded antimicrobial compounds. Njenga et al. isolated actinobacteria from an acid mine drainage (AMD) biofilm which were screened for antibiotic activity against *Staphylococcus aureus* and *Escherichia coli* (2025). An acidophilic *Nocardiopsis* strain secreted a pigmented angucycline polyketide with strong antibacterial activity at low pH, potentially enabling niche dominance within the biofilm (Njenga et al., 2025). Another isolate from the same environment produced a tripyrrole antibiotic resembling prodigiosin, which retained activity even in 100 mM Fe^2+^ medium—a condition under which most conventional drugs are inactivated. These discoveries suggest that extreme acidophiles are promising sources of chemically resilient antibiotics.

Extremophile-derived antimicrobials have also been isolated from thermal habitats. In microbial mats from a UV-exposed, nutrient-poor hot spring in the Atacama Desert (~45 °C), *Bacillus* sp. LB7 and *Streptomyces* sp. LB8 were found to produce multiple antimicrobial metabolites (Pardo-Esté et al., 2024). Genome mining revealed a biosynthetic gene cluster encoding huascopeptin-1, a novel class II lasso peptide active against *Bacillus subtilis*. *Bacillus* LB7 secreted distinct antimicrobial compounds at both 37 °C and 58 °C, with chemical analyses linking them to flavone and myxalamide analogs (Pardo-Esté et al., 2024; Shamsudin et al., 2022). The thermostability and broad activity of these compounds likely contribute to the community’s resistance to invasion, highlighting the potential of extremophilic biofilms to yield structurally novel and clinically relevant secondary metabolites. In clinical contexts, a few compounds derived from organisms with extremotolerant characteristics show promise. Cold-Azurin, a cold-adapted blue copper protein from an Antarctic *Pseudomonas* sp., has been shown to inhibit *Staphylococcus epidermidis* biofilm formation on surfaces (D’Angelo et al., 2024). Recombinant production in *E. coli* has already been achieved, suggesting future use as a coating for medical implants or catheters to prevent infection in clinical settings (D’Angelo et al., 2024). Other examples include kribbellichelins A and B, two antibiotics derived from a halophilic *Kribbella* sp. in a Spanish saline wetland, which exhibit activity against a range of human pathogens (Virués-Segovia et al., 2022). Together, these findings demonstrate the potential of biofilms formed by extremophilic and extremotolerant microorganisms could be valuable reservoirs of chemically distinct antimicrobials, although translation into clinical testing and development for these compounds has largely yet to be pursued.

### 3.2 Antioxidants

Extreme environments are well-known sources of oxidative stress, typically taking the form of intense ultraviolet (UV) radiation, high salinity, and heavy-metal contamination. Biofilm-derived EPS have shown considerable antioxidant properties that protect against such conditions (Ali et al., 2020). This activity results from hydroxyl, carbonyl, and carboxyl functional groups in microbial exopolysaccharides that can donate electron pairs and neutralize reactive oxygen species (ROS). An α-mannan exopolysaccharide from the EPS of a psychrophilic *Sphingobacterium* sp. IITKGP-BTPF3 exhibited significantly higher superoxide radical scavenging activity than ascorbic acid at concentrations of ≥4 mg/mL for both compounds (both compounds are 50% effective at 0.5 mg/mL), despite demonstrating inferior DPPH, ABTS, and ferric reducing power, suggesting selective antioxidant potential (Chatterjee et al., 2018). The bacterium also demonstrated immunomodulatory capabilities by decreasing nitric oxide production in lipopolysaccharide-elicited murine macrophage cells, highlighting its potential as a therapeutic agent for managing oxidative stress and inflammatory responses (Chatterjee et al., 2018). Similarly, carboxymethylated sulfated EPS derived from a haloarchaeal strain exhibited significant scavenging activities against hydroxyl and superoxide radicals and prevented linoleic acid peroxidation, indicating its antioxidant potential and suitability for industrial and biomedical applications (Chouchane et al., 2020). Chouchane et al. also suggested that sulfate groups as a component of the EPS play an important role in free radical scavenging and could perhaps be exploited in novel antioxidant designs.

Lin et al. characterized DeinoPol, an exopolysaccharide produced by the extreme radiation resistant *Deinococcus radiodurans* that plays a significant role in bacterial attachment and biofilm development in these environments (2020). DeinoPol exhibited a significant reduction in intracellular ROS levels in human keratinocytes (HaCaT) when exposed to UVB radiation (120 mJ/cm^2^), reducing ROS-induced cell death and apoptosis (Lin et al., 2020). DeinoPol also enhanced wound healing by protecting cells from ROS-induced damage and promoting cell migration and proliferation while protecting against γ-irradiation, hydrogen peroxide, and desiccation. This suggested that the bacterium’s EPS, and that of radiation-resistant bacteria more broadly, could serve as highly effective antioxidants, radioprotectants, and potentially even medical therapies.

Other studies have investigated exopolysaccharide-producing extremophiles and confirmed many of their potent antioxidant capabilities, including those from the halophilic bacteria *Halolactibacillus miurensis* and *Haloterrigena turkmenica* (Arun et al., 2017; Zhao et al., 2022; Squillaci et al., 2016), demonstrating similar dose-dependent scavenging capabilities against superoxide radicals. Indeed, beyond polysaccharides, pigments produced by these structures contribute to oxidative stress protection. Halophilic archaea produce bacterioruberin, a C₅₀ carotenoid localized in membranes and EPS sheaths (Hwang et al., 2024). Bacterioruberin and its derivatives are highly conjugated polyenes that quench singlet oxygen and stabilize peroxyl radicals (Giani et al., 2023; Hwang et al., 2024). Extracts from *Haloferax* species have shown stronger antioxidant activity than β-carotene or astaxanthin and remain stable at saturating salinity and temperatures up to 50 °C. *In situ*, these pigments protect haloarchaeal biofilms from solar radiation and peroxide stress (Hwang et al., 2024). In fact, bacterioruberin has been shown to be ~10× more effective than vitamin E in preventing oxidative DNA damage, with current research focused on optimizing its production (e.g., via *Haloferax* as a “C₅₀ carotenoid factory”) and enhancing its stability for biomedical and cosmeceutical applications. Other compounds, such as mycosporine-like amino acids (MAAs), UV-screening antioxidants from extremophilic algae and cyanobacteria, have already been successfully commercialized in products like Helioguard™365, offering eco-friendly protection against UV-induced oxidative stress. Together, these examples illustrate how extremophile-derived antioxidants—from carotenoids to small molecules—are not only essential for microbial survival but also hold translational potential in pharmaceuticals, cosmetics, and food preservation.

### 3.3 Anticancer agents

Cancer remains one of the most difficult illnesses to treat, often developing mechanisms to evade immune responses and resist various chemotherapeutic interventions (Anand et al., 2022). Intriguingly, biofilm-derived EPS exhibit diverse bioactivities, including anti-inflammatory and antitumor properties (Du et al., 2017; Giani et al., 2023). Giani et al. evaluated carotenoid-rich extract from *Haloferax mediterranei* biofilms, which exhibited dose-dependent cytotoxicity against several breast cancer cell lines, particularly triple-negative breast cancer (TNBC) (2023). The extract, composed primarily of bacterioruberin, reduced cancer cell viability to 12–65% at 100 μg/mL and had no significant effect on the viability, morphology, and diameter of non-cancerous mammary epithelial cells (184A1) even at the highest concentration tested (100 μg/mL) measured 30 h after treatment, suggesting selective cytotoxicity (Giani et al., 2023). Morphological changes consistent with apoptosis were observed in treated cells, and the bioactivity of the extract persisted under high salt and thermal stress, indicating high compound stability, which could be advantageous in drug development. The molecular mechanism for this selective cytotoxicity, while not yet fully elucidated, may stem from the ability of haloarchaeal carotenoids to induce apoptosis through caspase activation and to inhibit matrix metalloprotease-9 (MMP-9), a key mediator of tumor invasion and metastasis (Hegazy et al., 2020; Giani et al., 2023). Other carotenoids such as lutein have selectively increased ROS in cancer cells, particularly triple-negative subtypes, while sparing normal cells with more robust antioxidant defenses (Gong et al., 2018). While more experimental evidence will be needed to elucidate these compounds’ molecular mechanism and health benefits in humans, these studies position haloarchaeal pigments as potentially promising anticancer agents against aggressive drug-resistant cancers (Giani et al., 2023).

Cave-derived microorganisms have also yielded bioactive anticancer metabolites. A strain of *Nonomuraea* sp. PT708, isolated from Thai cave soil, exhibited selective cytotoxicity toward small-cell lung carcinoma (NCI-H187) and oral cavity cancer cell lines (KB) with IC₅₀ values of 3.48 and 16.11 μg/mL, respectively (Nakaew et al., 2009; Nakaew et al., 2012). At concentrations up to 50 μg/mL, the extract demonstrated no inhibitory effect on MCF7 breast cancer cells (Nakaew et al., 2009; Nakaew et al., 2012). This suggests that such compounds may exert activity specific to certain cancer types via distinct cellular pathways. In addition to small molecules, exopolysaccharides produced by extremotolerant biofilms have shown potential immunomodulatory and anticancer effects. The EPS from *Pseudomonas alcaligenes* Med1, isolated from a hot spring, stimulated macrophage cytokine production and inhibited proliferation of a human carcinoma cell line by ~30% in preliminary studies, indicating potential antitumor and immunoregulatory effects (Sarkar et al., 2024). If confirmed with more experimental data (e.g., IC₅₀ values on various cancer cells, apoptosis markers), this would add a new dimension to extremophile EPS as anticancer adjuvants.

The evolutionary mechanisms underlying the production of these compounds remain unclear. However, it is hypothesized that in extreme environments, EPS and other bioactive metabolites that inhibit the growth of competitors may confer a selective advantage with potential action on cancer cells, likely by acting antioxidants, scavenging hydroxyl and superoxide radicals, binding to carcinogens, and improving overall immunity (Nakaew et al., 2012; Homero et al., 2021). The translational precedent for compounds produced by extremophilic biofilms is less robust for anti-cancer agents compared to other applications; however, cold-adapted azurin peptides derived from extremotolerant *Pseudomonas* species have demonstrated cell-penetrating and p53-stabilizing properties (D’Angelo et al., 2024; Hu et al., 2023). Although initial trials involved mesophilic variants, psychrophilic forms may offer enhanced stability or activity, representing a potential future direction for extremophile-derived anticancer therapeutics.

### 3.4 Cryoprotectants

Cryoprotectants protect biological tissue from freezing damage and are produced by polar and alpine psychrophiles as an adaptive mechanism to extreme temperatures (D’Amico et al., 2006). EPS produced by bacteria to aid in biofilm formation also contribute to cryoprotection by inhibiting ice crystal growth and stabilizing proteins and membranes (Marx et al., 2009). Biofilm-forming psychrophiles have been primarily studied in the deep sea and glaciers but are found in nearly all habitats where psychrophiles reside, including polar soils, permafrost, and freshwater (D’Amico et al., 2006).

EPS isolated from an Antarctic marine *Pseudoalteromonas* sp. Roscoff strain RosPo-2, named “p-CY02,” demonstrated strong cryoprotective effects, including improved viability of mammalian cells after cryopreservation (Kang et al., 2024). Adding 0.8% p-CY02 to the freezing medium (alongside a low dose of DMSO) significantly enhanced survival of human keratinocyte cells (HaCaT) after liquid-nitrogen freezing: 87.9 ± 2.8% of cells remained viable with 5% DMSO + 0.8% EPS, which was 1.7-times greater than with 5% DMSO alone (Kang et al., 2024). This glycine-rich, high-molecular-weight EPS can reduce ice-induced cell damage, likely by forming protective matrices that suppress crystal growth and osmotic shock (Kang et al., 2024). Such polar bacterial EPS may offer a non-toxic cryoprotectant alternative for cell and tissue preservation.

For example, *Pseudomonas* sp. BGI-2, isolated from the Batura Glacier, secretes viscous EPS that significantly reduces freeze-induced lysis in human red blood cells and improves bacterial survival rates under freezing stress (Ali et al., 2020). EPS from this and related strains have been shown to outperform or match conventional cryoprotectants such as glycerol (Ali et al., 2020). Chemical analysis has revealed polysaccharide compositions rich in glucose, galactose, and glucosamine, contributing to cryoprotection through viscosity modulation and microenvironment stabilization. In addition to EPS, several extremophilic microorganisms secrete antifreeze proteins (AFPs) into their biofilms (Białkowska et al., 2020; Lopes et al., 2024). These proteins inhibit ice recrystallization and contribute to biofilm integrity under freezing conditions. One such protein, cold-azurin, produced by an Antarctic *Pseudomonas* strain, has been shown to prevent ice damage in model multispecies biofilms (D’Angelo et al., 2024). These findings show that extremophiles deploy both sugar-based cryoprotectants and specialized proteins to endure freezing conditions.

Notably, compatible solutes such as ectoine and hydroxyectoine—cyclic amino acid derivatives synthesized by halophilic bacteria like *Halomonas elongata*—have shown efficacy in stabilizing proteins and membranes under cold conditions (Sun et al., 2012). Some of these compounds improve post-thaw cell viability and have progressed to commercialization for different applications. Ectoine, for instance, is produced at industrial scale and has been incorporated into bioprotective medical products, including nasal sprays for mucosal hydration (Chen et al., 2023). Clinical studies have demonstrated its efficacy and safety, further supporting its translational potential in cryopreservation and tissue protection (Kauth and Trusova, 2022). Antifreeze proteins and EPS derived from psychrophilic bacteria and algae are also under preclinical investigation for applications in biobanking and organ preservation (Ekpo et al., 2022; Baskaran et al., 2022). While no extremophile-derived antifreeze protein has yet reached the market, these data suggest a promising future for future biomedical and biotechnological deployment.

### 3.5 Bioremediation

Bioremediation is the process of using living organisms, particularly microbes, to degrade, neutralize, or remove pollutants from the environment (Sonawane et al., 2022). Here, we discuss bioremediating compounds due to their implications in larger biological systems. Because biofilm EPSs are rich in carboxyl, phosphate, hydroxyl, and sulfhydryl groups that electrostatically attract metal cations, they can be effective biosorbents for removing heavy metals, radionuclides, and other pollutants (Sonawane et al., 2022). From an economic and feasibility standpoint, biofilms are also considered a promising solution in industry, as they do not need to be separated from the bulk liquid waste (Dalahmeh et al., 2019).

Extremophilic biofilms use a combination of biosorption (surface binding), bioreduction (redox detoxification), and bioprecipitation (mineral formation) to remove and immobilize contaminants (Ayilara and Babalola, 2023). The two primary application targets are aqueous systems (e.g., industrial wastewater, nuclear effluent) and soil systems (e.g., mining or chromate-contaminated sites). In water-based reactors, microbial EPS binds dissolved metal ions and promotes their aggregation into biomass-associated granules or insoluble mineral phases. These aggregated forms have higher density and larger size than free ions or colloids, enabling easier removal by sedimentation, filtration, or backflushing (Ayilara and Babalola, 2023). In soil environments, microbial biofilms typically operate *in situ* by converting soluble toxins into less mobile mineral forms (e.g., metal sulfides or phosphates), thereby preventing further leaching and reducing bioavailability (Ayilara and Babalola, 2023; Misra et al., 2024). Across both systems, biofilms function as biological concentrators, sequestering and localizing contaminants within defined biomass structures, and making recovery and isolation far more tractable than when contaminants are freely dispersed in water or loosely adsorbed to soil matrices.

In alkaline saline wastes, for example, haloalkaliphilic bacteria reduce Cr(VI) to the less toxic and less mobile Cr(III), which is then immobilized within the biofilm matrix (Martínez-Espinosa, 2024; Varshney et al., 2023). *Lysinibacillus mangiferihumi*, isolated from chromate-contaminated alkaline soil, reduces Cr(VI) enzymatically and sequesters Cr(III) via EPS-mediated binding, making the contaminant easier to isolate from the environment (Tambekar et al., 2015). While not a prokaryote, the acidophilic alga *Galdieria sulphuraria* forms biofilms at pH 1–2 and 45 °C and biosorbs cadmium and lead through its polysaccharide-rich cell envelope, which contains metal-chelating groups such as carboxylates and sulfates (Kharel et al., 2023). In soil remediation, the precipitated Cr(III) or Cd/Pb minerals remain immobilized *in situ*, reducing leaching and bioavailability (Varshney et al., 2023). In aqueous systems such as wastewater, Cr-containing biofilm, for example, form millimeter-scale granules that settle 5–10× faster than colloidal Cr, allowing gravity-sedimentation or lamella clarifiers to harvest metal-rich sludge for safe disposal or metal recovery (Martínez-Espinosa, 2024; Varshney et al., 2023; Anabtawi et al., 2025).

Radioresistant extremophiles demonstrate in-situ radionuclide immobilization (Roy et al., 2023). *Deinococcus radiodurans* biofilms grown on glass beads remove ~90% of dissolved U(VI) from simulated nuclear wastewater by reducing it to insoluble U(IV) and trapping the precipitate within phosphate-rich EPS (Manobala et al., 2021). EPS-associated phosphorylated sugars facilitate uranium binding (Manobala et al., 2021). The resulting U-laden biofilms can be removed by backflushing the reactor to dislodge EPS-bound U(IV) into a concentrated sludge, enabling safe and efficient separation of radioactive waste from the treated water (Manobala et al., 2021). Similarly, sulfate-reducing bacteria (SRB) in underground biofilms near radioactive waste repositories precipitate heavy metals and radionuclides as sulfides, immobilizing them (Ruiz-Fresneda et al., 2023). These SRB operate at pH ~ 11 and high calcium—extreme conditions found in cementitious nuclear waste leachate—yet their biofilms flourish and continually sequester Strontium-90 and Cesium-137 into stable mineral forms (Ruiz-Fresneda et al., 2023). In a continuous-flow bioreactor system, SRB consortium immobilized on polyethylene biofilm carriers achieved 99–100% removal of dissolved lead from synthetic wastewater containing 100–150 mg L^−1^ Pb within 40 days of operation, with a hydraulic retention time of 5 days (Quynh et al., 2015). Lead was precipitated as insoluble PbS and retained within the biofilm matrix, forming sludge that remained stably sequestered throughout the treatment period, (Quynh et al., 2015). These results underscore the viability of SRB biofilms for continuous *in situ* heavy metal treatment without the need for soil excavation or frequent sludge removal (Quynh et al., 2015).

EPS produced from a marine *Pseudomonas furukawaii* demonstrated the ability to emulsify and degrade up to 89.5% of crude oil within 5 days under simulated marine spill conditions, outperforming *P. furukawaii* cells alone (67.8%) (Vandana and Das, 2021). In practice, these emulsified oil droplets remained suspended and could be recovered by solvent extraction or surface skimming. Notably, the EPS maintained stability at 85 °C and showed no phase separation after 15 days, highlighting its resilience in high-salinity, thermally variable marine environments—this, combined with EPS’ emulsification ability, give them tremendous potential for bioremediation of oil polluted marine sites (Camacho-Chab et al., 2013; Vandana and Das, 2021). Similarly, thermotolerant EPS-producing *Pseudomonas* sp. W6 isolated from an Indian hot spring chelated ~62% of lead (Pb) from wastewater in a pilot treatment setup in 12 h (Kalita and Joshi, 2017).

These results begin to highlight the potential of biofilm-derived polymers for use in industrial applications including wastewater treatment, mining effluent remediation, and oil spill cleanup. Several of these systems are currently undergoing field trials or advanced laboratory evaluation; *Acidithiobacillus* are already employed in bioleaching operations (do Nascimento et al., 2022), and several patents exist on extremophilic EPS for heavy-metal bioremediation and oil recovery (Banerjee et al., 2021). Collectively, these findings underscore the potential viability of extremophile-derived EPS as environmentally robust agents for bioremediation.

## 4 Functions and translational potential of extremophilic biofilms

Extremophilic biofilms have emerged as reservoirs of functionally diverse and resilient biomolecules. Bacterioruberin-rich extracts from *Haloferax mediterranei* biofilms appear to exhibit selective cytotoxicity against TNBC, α-mannan EPS from Arctic *Sphingobacterium* sp., demonstrates superoxide scavenging activity exceeding that of ascorbic acid, EPS matrices from *Deinococcus radiodurans* sequester ~90% of uranium as U(IV), and *Lysinibacillus mangiferihumi* can reduce and immobilize Cr(VI) in alkaline, chromate-contaminated soils—among a few examples of the bioactivities of biofilm-derived compounds in extreme environments (Table 1) (Giani et al., 2023; Chatterjee et al., 2018; Kang et al., 2024; Chen et al., 2021; Manobala et al., 2021).

Some of the compounds described in this review fall under multiple categories of bioactivity (Table 1). For instance, Antarctic-derived EPS have been shown to exhibit cryoprotective, antioxidant, and bioremediating effects (Chatterjee et al., 2018; Nagar et al., 2021; Caruso et al., 2017). The evolutionary basis for EPS multifunctionality in extreme environments is not completely understood. However, there is evidence to suggest that certain EPS chemistries may confer selective advantages under specific environmental stresses—acidophilic taxa often secrete uronic acid-enriched polymers that chelate metal ions and buffer local pH, while psychrophiles synthesize glycine- and sulfate-rich EPS that inhibit ice recrystallization and maintain hydration under subzero temperatures (Moncayo et al., 2022; Casillo et al., 2017). This conservation of general EPS features and compounds, coupled with variability in monosaccharide composition, degree of sulfation, and glycine content, may enable extremophiles to support a broad range of biochemical functions while still containing extracellular matrices that are useful for their niche-specific stresses (Caruso et al., 2017). Furthermore, certain molecules appear to confer multiple bioactivities: in *Haloferax* biofilms, the carotenoid bacterioruberin appears to confer antioxidant and antitumor effects, all while remaining stable under high salt and thermal stress (Table 1) (Giani et al., 2023; Hwang et al., 2024; Ali et al., 2020). Cold-adapted azurin from an Antarctic *Pseudomonas* sp. TAE6080 inhibits *Staphylococcus epidermidis* biofilm formation on abiotic surfaces, exhibits antitumor properties through p53 stabilization and caspase activation, and simultaneously has cryoprotective effects (D’Angelo et al., 2024; Hu et al., 2023). The evolutionary drivers underlying these diverse bioactivities remain unknown, but the functional breadth of these compounds hold translational potential (Graça et al., 2016; Murray et al., 2024; Madsen et al., 2016).

Despite the robust array of such compounds that have demonstrated promising bioactivities *in vitro,* efforts to translate them into applied contexts for therapeutic purposes have remained nascent (Chattopadhyay et al., 2022). For instance, nearly all the non-remediating compounds listed in Table 1 are still in the research or discovery phase with limited or no attempts at downstream development. This stands in stark contrast to the widespread pharmaceutical success of antimicrobials derived from mesophilic soil organisms; most notably, the *Streptomyces* genus, which has yielded numerous clinically approved antibiotics (Quinn et al., 2020). This is evidenced by the fact that many reported bioactivities are attributed to partially characterized extracts or polymer fractions, often lacking full structural elucidation or mechanistic understanding (Homero et al., 2021; Giani et al., 2023; Nakaew et al., 2012). For instance, many EPS-associated activities have been assessed only in crude form, with little insight into the precise branching patterns or protein conjugates responsible for function (Giani et al., 2023; Nakaew et al., 2012). Cultivation challenges further complicate this landscape. Many extremophiles are difficult to grow under laboratory conditions, and replicating the in-situ expression levels of their metabolites remains elusive, owing in part to the limited understanding of the ecological roles and environmental triggers that regulate their biosynthesis (Rampelotto, 2024; Schultz et al., 2023; Merino et al., 2019). As a result, very few extremophile-derived compounds have progressed to in-vivo testing or been evaluated for key pharmacological properties such as pharmacokinetics, bioavailability, or toxicity (Gallo and Aulitto, 2024). Thus, the underdevelopment of extremophile-derived metabolites stems not from a lack of biochemical promise, but from a confluence of scientific, technical, and translational barriers that have historically hindered their advancements beyond the bench.

Despite the challenges outlined above, we remain optimistic about the translational potential of extremophilic biofilm-derived compounds. These molecules, shaped by evolution to withstand extreme physicochemical stressors, possess inherent resilience that could prove valuable in a wide range of physiological and industrial contexts (Yin et al., 2019; Parrilli et al., 2022). Recent methodological advances further strengthen this outlook. Improved techniques for selective isolation and cultivation of extremophiles—including optimized ichip diffusion chambers, high-pressure and temperature-stable sampling containers, and specialized *in situ* enrichment strategies—are expanding access to previously unculturable taxa (Gallo and Aulitto, 2024; Zhao et al., 2023). Moreover, targeted bioprospecting of underexplored extreme environments, such as subglacial lakes, alkaline volcanic springs, and high-radiation desert soils, could hold promise for uncovering novel biosynthetic gene clusters with unique bioactivities. The integration of long-read metagenomics and single-cell sequencing now makes it feasible to recover and characterize these clusters even from uncultivated or low-abundance taxa (Liu et al., 2022). These tools, paired with advances in synthetic biology, provide a path forward for expressing and optimizing such clusters in heterologous hosts (Gallo and Aulitto, 2024). Structure-guided compound engineering could also be the key to unlocking new applications. Lead candidates such as cold-adapted azurin variants or thermostable lasso peptides could be systematically modified to improve pharmacokinetics, production efficiency, and therapeutic index (D’Angelo et al., 2024; Yaghoubi et al., 2020; Xiu et al., 2022). Similarly, exopolysaccharides and antifreeze proteins with demonstrated biophysical resilience offer templates for new biomaterial development, including cryopreservatives, wound dressings, and hydrogels (Lopes et al., 2024).

## 5 Conclusion

- There are five major classes of bioactive compounds derived from extremophilic biofilms, including antimicrobials, antioxidants, cryoprotectants, anticancer agents, and bioremediation tools. These have potential efficacy across a range of biomedical and environmental contexts (Table 1).
- Several extremophilic EPS and metabolites fall under multiple bioactivity classes. The evolutionary drivers of this multifunctionality remain unknown; however, their broad range of functions holds potential in therapeutic and industrial contexts.
- Despite this promise, most compounds remain in the early research stage. Cultivation challenges, incomplete structural elucidation of EPS, and limited understanding of ecological triggers regulating metabolite expression have likely hindered downstream development. However, methodological advances are beginning to overcome some of these barriers. Long-read metagenomics, single-cell sequencing, and synthetic biology could provide tools to recover biosynthetic clusters from previously uncultivable taxa, while structure-guided engineering offers opportunities to optimize stability, pharmacokinetics, and production yields.
- Considering the urgent need for novel antimicrobials, sustainable biotechnologies, and climate-resilient materials, we advocate for more systematic exploration and sustained investment into the biology, chemistry, and engineering of extremophilic biofilms. Looking ahead, cross-disciplinary efforts that connect microbial ecology, structural biology, and translational research will be important for advancing this field. We hope this review will spur deeper mechanistic investigation into the regulation and function of these metabolites *in situ*, work that will be essential for harnessing their full clinical and biotechnological potential.
